# Whole Cigarette Smoke Condensates Induce Accumulation of Amyloid Beta Precursor Protein with Oxidative Stress in Murine Astrocytes

**DOI:** 10.3390/toxics9070150

**Published:** 2021-06-28

**Authors:** Eun-Jung Park, Seung-Woo Jin, Hyun-Ji Lim, Hyeon-Young Kim, Min-Sung Kang, Siyoung Yang

**Affiliations:** 1 East–West Medical Science Research Institute, Kyung Hee Medical Science Research Institute, Kyung Hee University, Seoul 02447, Korea; 2 Human Health and Environmental Toxins Research Center, Kyung Hee Medical Science Research Institute, Kyung Hee University, Seoul 02447, Korea; 3 Department of Biomedical Science and Technology, Graduate School, Kyung Hee University, Seoul 02447, Korea; wlstmddn@khu.ac.kr (S.-W.J.); dlaguswl1001@khu.ac.kr (H.-J.L.); mskang@kitox.re.kr (M.-S.K.); 4 Inhalation Toxicology Center for Airborne Risk Factors, Jeonbuk Branch Institute, Korea Institute of Toxicology, Jeongeup 56212, Korea; hyeonyoung.kim@kitox.re.kr; 5 General Toxicology & Research Group, Jeonbuk Branch Institute, Korea Institute of Toxicology, Jeongeup 56212, Korea; 6 Department of Pharmacology, Ajou University School of Medicine, Suwon 16499, Korea; yangsy@ajou.ac.kr; 7 Degenerative InterDiseases Research Center, Ajou University School of Medicine, Suwon 16499, Korea

**Keywords:** cigarette smoking, Alzheimer’s disease, astrocytes, autophagy, immune response

## Abstract

Although cigarette smoking has been postulated to be a potential risk factor for Alzheimer’s disease (AD), the toxic mechanism is still unclear. Additionally, astrocytes have been identified as a potential target, given they play multiple roles in maintaining normal brain function. In this study, we explored the toxic mechanism of whole cigarette smoke condensates (WCSC) using murine astrocytes. Cell proliferation, the percentage of cells in the G2/M phase, and LDH concentrations in the cell supernatants were all reduced in WCSC-treated cells. In addition, oxidative stress was induced, together with shortening of processes, structural damage of organelles, disturbances in mitochondrial function, blockage of autophagic signals, accumulation of amyloid β precursor protein, and loss of chemotactic functions. Based on these results, we hypothesize that dysfunction of astrocytes may contribute to the occurrence of cigarette-smoking-induced AD.

## 1. Introduction

Dementia is the fifth leading cause of death worldwide and affects 50 million people. Although many potential causes, including physical, psychological, social, and economic circumstances, are associated with the incidence of dementia, it is clear that age is the most significant risk factor [1]. Thus, the trend toward aging populations and the progressive increase in the number of patients with dementia have become worldwide concerns. In addition, Alzheimer’s disease (AD) is the most frequent form of dementia and accounts for 60–70% of all dementia cases [2]. Since the identification of amyloid plaques in the brains of AD patients, several studies have reported that the gradual accumulation of misfolded or aggregated amyloid-beta (Aβ) protein may greatly contribute to the pathogenesis of AD [3,4,5].

Cigarette smoking has been considered an important risk factor for AD, suggesting the possible prevention (or delay) of AD onset (or progress) by quitting smoking [6,7,8]. The risk of AD was reported to be 2.56-fold greater among medium-level smokers, compared with nonsmokers (15–24 cigarettes per day) [7,9]. In addition, oxidative stress increased amyloid β precursor protein (APP) processing and microglial proinflammatory responses, and reduced Aβ clearance by microglia are also closely associated with cigarette-smoking-induced AD.

Glial cells, including microglia and central-nervous-system (CNS)-resident cells, comprise about 90% of human brain cells, and they have been known to play an important role in the immune response to protect the CNS against stress and pathogens [10]. Particularly, astrocytes are the most abundant, comprising approximately 30% of the cells in the CNS, and can fuel neuronal growth through gluconeogenesis when there is an urgent glucose demand in the brain [11]. Increasing evidence also shows that astrocytes act as key players in the brain, as they are involved in the uptake or release of neurotransmitters, maintenance of the blood–brain barrier, regulation of ion homeostasis in the extracellular space and blood, nervous system repair, and signal transduction [12,13,14]. More importantly, astrocytes function as antigen-presenting cells for T cell activation in immune responses to xenobiotics and pathogenic microorganisms entering the CNS [15,16]. Therefore, astrocyte impairment may cause overall brain dysfunction, ultimately leading to various neurodegenerative diseases, including AD and Parkinson’s disease [14,17,18].

Previously, we have demonstrated that whole cigarette smoke condensates (WCSC) contain many constituents including chemicals (hydrophilic and hydrophobic), particulate matters, and gaseous and that WCSC induces ferroptosis via ER stress, disturbance of mitochondrial dynamics, and activation of the hypoxia-inducible factor-1 pathway in human bronchial epithelial cells [19]. In addition, defective ferroptotic cell death is linked to tumorigenesis [20], and an inverse relationship between the onset of cancer and AD has been demonstrated by some epidemiological studies [21,22,23,24,25]. Therefore, we aimed to identify the underlying mechanisms of WCSC-induced neurotoxicity using murine astrocytes.

## 2. Materials and Methods

### 2.1. Cell Culture 

The WCSC was kindly provided by Dr. Kyu-hong Lee, and murine astrocytes (C8-D1A) were purchased from the American Type Culture Collection (Manassas, VA, USA) and maintained in complete DMEM medium supplemented with 10% (*v*/*v*) fetal bovine serum, 100 U/mL penicillin, and 100 μg/mL streptomycin (ThermoFisher Scientific, Waltham, MA, USA) under a humidified atmosphere containing 5% CO_2_/95% air at 37 °C [26].

### 2.2. Cell Viability Assays

Cells (2 × 10^4^ cells/mL) were stabilized overnight on a 96-well plate and treated with WCSC (0, 0.25, 0.5, or 1%) for 24 h. The MTT solution (Sigma-Aldrich, St. Louis, MO, USA) was added to each well and the cells were incubated for 4 h at 37 °C. Then, formazan crystals were dissolved with dimethyl sulfoxide (Duchefa Biochemie, Haarlem, RV, Nederland), and the absorbance was measured at 540 nm using a multimode microplate reader (BioTek, Winooski, VT, USA).

### 2.3. Cell Cycle Analysis

Under a phase-contrast microscope, we found that cell proliferation was reduced by WCSC treatment; however, dead cells were not evident at up to the maximum concentration tested. Thus, we investigated changes in the cell cycle following exposure to WCSC. After 24 h exposure to WCSC (0, 0.25, 0.5, or 1%), the cells were washed once with phosphate-buffered saline and fixed in 70% ethanol overnight at 4 °C. The cells were then incubated with RNase (100 μg/mL) for 1 h at 37 °C, and propidium iodide (50 μg/mL) was added to each sample. Finally, the cells were analyzed using a flow cytometer (FACSAria III, BD Biosciences, Franklin Lakes, NJ, USA).

### 2.4. Transmission Electron Microscopy (TEM)

Cells (60–70% confluency) were incubated in a culture medium containing 1% WCSC for 24 h and washed with PBS. The cells were fixed overnight with Karnovsky’s fixative solution and followed by 1% osmium tetroxide. The cells were stained with 0.5% uranyl acetate for 30 min, dehydrated using graded ethanol series (30, 50, 70, 80, 90, and 100%), and passed through propylene oxide. The cells were then embedded in Spurr’s resin for 24 h at 70 °C, and ultrathin sections were placed on a copper grid and examined under a transmission electron microscope (TEM, Talos L120C, FEI, Prague, Czech Republic).

### 2.5. Measurement of Intracellular Reactive Oxygen Species

Intracellular reactive oxygen species (ROS) level was evaluated using carboxy-2′,7′-dichlorofluorescein-diacetate (H_2_DCFDA, Invitrogen, Waltham, MA, USA). Briefly, cells (60–70% confluency) were incubated with WCSC (0, 0.25, 0.5, or 1%) for 24 h and further incubated in an FBS-free culture medium containing H_2_DCFDA (10 μM) for 30 min at 37 °C. After washing once with PBS, the cells were resuspended in the FBS-free medium, and intracellular fluorescence intensity (488 nm) was analyzed using a flow cytometer (BD Biosciences).

### 2.6. Effects on Organelle Structure and Function

Effects on the integrity of the plasma membrane were evaluated using a commercially available lactate dehydrogenase (LDH) assay kit (Cat No. 88954, ThermoFisher Scientific). Briefly, cells (1 × 10^6^ cells/mL) were incubated in a 96-well plate with a designated concentration of WCSC for 24 h, and an aliquot of the supernatant (50 μL/well) was reacted with LDH reaction solution (50 μL/well) in a new 96-well plate for 30 min at RT. The absorbance of the reactants was quantified at 450 nm. Additionally, cells exposed to WCSC (0, 0.25, 0.5, or 1%), were incubated with MitoTracker Green FM (150 nM, for mitochondrial mass), MitoTracker™ Red (200 nM, for mitochondria membrane potential), MitoTracker™ Deep-Red FM (100 nM, for detection of active mitochondria), Rhod-2 (AM, 1 μM, for detection of mitochondrial calcium ions), ER Tracker™ Green (250 nM, for ER volume), or LysoTracker™ Green (50 nM, for lysosomes) according to the manufacturer’s instruction (Molecular Probes, Eugene, OR, USA). After washing once with PBS, intracellular fluorescence intensity was measured using a flow cytometer (BD Biosciences). Cells (2 × 10^4^ cells/well) were also stabilized in a 96-well white plate overnight. After 24 h incubation with WCSC (0, 0.25, 0.5, or 1%), CellTiter-Glo^®^ reagent (Promega, Fitchburg, WI, USA) was added to each well (200 μL/well), and the mixture reacted at RT for 10 min. Produced total ATP content was calculated by measuring luminescence values using a multimode microplate reader (BioTek, Winooski, VT, USA).

### 2.7. Gene Expression

Microarray analysis and polymerase chain reaction (PCR) tests were performed to identify changes in gene profiles and concentration-dependent changes in the expression of specific genes. First, total RNA was extracted using a TRIzol™ reagent (Invitrogen), and RNA purity and integrity were evaluated using a UV–Vis spectrophotometer (ND-Lite, ThermoFisher Scientific) and Agilent 2100 bioanalyzer (Agilent Technologies, Palo Alto, CA, USA). For microarray analysis (Macrogen, Seoul, Korea), Affymetrix^®^ whole-transcript expression array process was executed according to the manufacturer’s protocol (GeneChip^®^ Whole Transcript PLUS Reagent Kit, ThermoFisher Scientific). Briefly, cDNA was synthesized using a GeneChip^®^ Whole Transcript Amplification kit, as described by the manufacturer. The sense cDNA was then fragmented and biotin-labeled with TdT (terminal deoxynucleotidyl transferase) using a GeneChip^®^ WT Terminal labeling kit. Approximately 5.5 μg of labeled target DNA was hybridized to the Affymetrix^®^ GeneChip^®^ Mouse 2.0 Array at 45 °C for 16 h. Hybridized arrays were washed, stained on a GeneChip^®^ Fluidics Station 450, and scanned on a GCS3000 Scanner (ThermoFisher Scientific). Signal values were computed using Affymetrix GeneChip^®^ Command Console^®^ software. The data were summarized and normalized with the robust multi-average (RMA) method implemented in Affymetrix^®^ Power Tools. We exported the results for gene-level RMA analysis and performed differentially expressed gene analyses. Gene enrichment and functional annotation analyses for a significant probe list were performed using Gene Ontology (www.geneontology.org/, accessed on 24 June 2021) and Kyoto Encyclopedia of Genes and Genomes (www.genome.jp/kegg/, accessed on 24 June 2021). All data analyses and visualization of differentially expressed genes were conducted using R 3.3.2 (www.r-project.org, accessed on 24 June 2021). Amplified cDNA products were also produced using an AccuPower^®^ RT-PCR and PCR PreMix tubes (Bioneer, Daejeon, Korea), according to the manufacturer’s instructions. The products were separated on a 1.5% agarose gel and visualized using a ChemiDoc™ XRS+ system (Bio-Rad Laboratories Inc., Berkeley, CA, USA). Table 1 shows the primer sequence.

### 2.8. Protein Expression

Homogenized cell lysates were centrifuged at 13,000 rpm for 30 min, and equal amounts of protein were separated by SDS–PAGE and then transferred to nitrocellulose membranes (HybondECL, Amersham Pharmacia Biotech, NJ, USA). The membranes were blocked with 5% skim milk in PBS containing 0.05% Tween-20 (PBST) for 1 h at room temperature RT. Then, the membranes were immunoblotted with primary antibodies [1:1000, phosphorylated (p)-extracellular signal-regulated kinase-1 (p-ERK; Cell Signaling Technology, Danvers, MA, USA); p62, caspase-1, receptor-interacting serine/threonine kinase (RIP)1, and RIP3 (Abcam, Cambridge, UK); amyloid β precursor protein (APP) and β-actin (Santa Cruz Biotechnology, Dallas, TX, USA)] overnight at 4 °C, followed by incubation with horseradish–peroxidase-conjugated secondary antibodies for 1 h at RT. The blotted bands were visualized using a ChemiDoc™ XRS+ system (Bio-Rad Laboratories, Inc. Hercules, CA, USA) and quantified using Image J software (National Institutes of Health, Bethesda, MD, USA). In addition, cells (1 × 10^4^ cells/well) were stabilized overnight on cover slides in a 12-well plate and incubated with or without 1% WCSC for 24 h. The cells were fixed with 4% formaldehyde and ice-cold methanol. The cells were blocked with 3% BSA in PBST for 1 h and reacted overnight at 4 °C with antibodies against APP, p62, Mitofusin (MF)1 and MF2 (Abcam, Cambridge, UK); Golgin97 (Santa Cruz Biotechnology) and Calnexin (Cell Signaling). After washing twice with PBST, the cells were incubated with Alexa-Fluor™-555- or -488-conjugated anti-IgG antibodies (Molecular Probes) for 2 h at RT and mounted using a mounting medium with DAPI (ImmunoBioScience Corp., Mukilteo, WA, USA). Finally, the cells were visualized using a confocal laser scanning microscope (LSM710, Carl Zeiss, Germany) installed at the National Center for Inter-University Research Facilities at Seoul National University.

### 2.9. ELISA

Cells (1 × 10^6^ cells/mL) were incubated in a 12-well plate with WCSC (0, 0.25, 0.5, and 1%) for 24 h. The concentrations of interleukin (IL)-1 beta (β), IL-6, tumor necrosis factor-alpha (TNF-α), chemoattractant protein-1 alpha (MCP-1α; eBioscience, San Diego, CA, USA), and chemokine (C-X-C motif) ligand 1 (CXCL1; R&D Systems, Minneapolis, MN, USA) in the supernatants was measured according to the manufacturer’s protocols. Finally, the absorbance was measured at 450 nm using a multimode microplate reader (BioTek), and the absolute values were calculated using standard curves produced under the same conditions. 

### 2.10. FACS Analysis

Cells (70–80% confluency) were exposed to WCSC (0, 0.25, 0.5, or 1%) for 24 h. Harvested cells were blocked with an anti-cluster-of-differentiation (CD)16/CD32 antibody (eBiosciences, San Diego, CA, USA). Then, the cells were incubated for 30 min at 4 °C with fluorochrome-conjugated anti-CD54, -MHC class II, and -CXCR2 (eBiosciences) antibodies, and the cell surface expression levels were analyzed using a flow cytometer (BD Biosciences).

### 2.11. Statistical Analyses

As previously reported, the statistical significance of the microarray data was determined using an LPE test and the fold change was determined using a null hypothesis of no difference among groups. The false-discovery rate was controlled by adjusting *p*-values using the Benjamini–Hochberg algorithm. For a DEG set, hierarchical cluster analysis was performed using complete linkages and Euclidean distances as measures of similarity. In addition, Student’s *t*-test (Prism 7, GraphPad Software, San Diego, CA, USA) and one-way ANOVA, followed by Tukey’s post hoc pairwise comparisons were used to determine the statistical significance of all other data.

## 3. Results

### 3.1. Reduced Cell Proliferation

**Figure 1:**
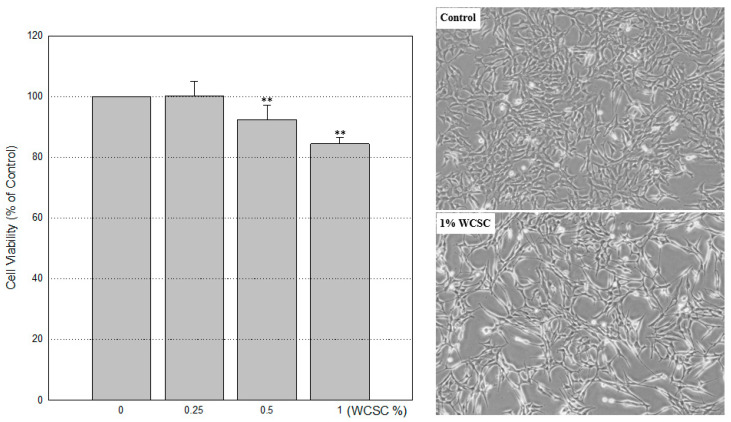
Decreased cell proliferation. Cell proliferation was assessed using the MTT assay, and the effects of WCSC treatment were calculated based on the absorbance of the control (100%). Experiments were independently performed three times using four wells per concentration. Data are the mean ± standard deviation (SD). ** *p* < 0.01. Pictures on the right show the decreased cell proliferation. Magnification: ×100.

**Figure 2:**
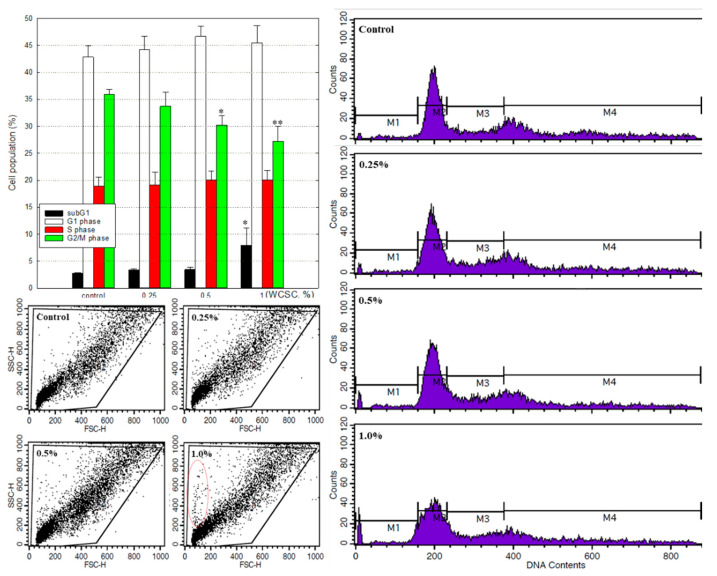
Imbalanced cell cycle regulation. Astrocytes were treated with WCSC in designated concentrations for 24 h. Treated and control cells were harvested, washed with PBS, and fixed in 70% ethanol overnight. After centrifugation, the cells were washed once with PBS and incubated in PBS containing 50 μg RNase for 1 h at 37 °C, and the cells were then stained with 5 μg PI. Experiment was independently performed three times (mean ± SD). * *p* < 0.05, ** *p* < 0.01.

After 24 h exposure to WCSC, no features consistent with those of dead cells were evident under phase-contrast microscopy, whereas proliferation appeared to be inhibited. When incubated with 0.25, 0.5, or 1% WCSC, the proliferation level was 100.2 ± 4.8%, 92.4 ± 4.6% and 84.4 ± 2.1%, respectively, compared to control (Figure 1). Cell-cycle analyses showed that apoptotic cells tended to increase in a concentration-dependent manner, accompanied by a reduction of cells in the G2/M phase (Figure 2). Cells in the G1, S, and G2/M phases were 42.8 ± 2.2%, 18.9 ± 1.7%, and 35.9 ± 1.0%, respectively, in control cells, whereas they were 45.4 ± 3.2%, 20.1 ± 1.8%, and 27.2 ± 2.8%, respectively, in cells treated with 1% WCSC. Additionally, cells in the subG1 region were 2.8 ± 0.1% and 7.9 ± 3.3%, in the control and 1% WCSC-treated cells, respectively. Furthermore, a decrease in cell size and an increase of cellular complexity were detected in 1% WCSC-treated cells (Figure 2 Below, a red dotted line circle region).

### 3.2. Damaged Organelle Structures

**Figure 3:**
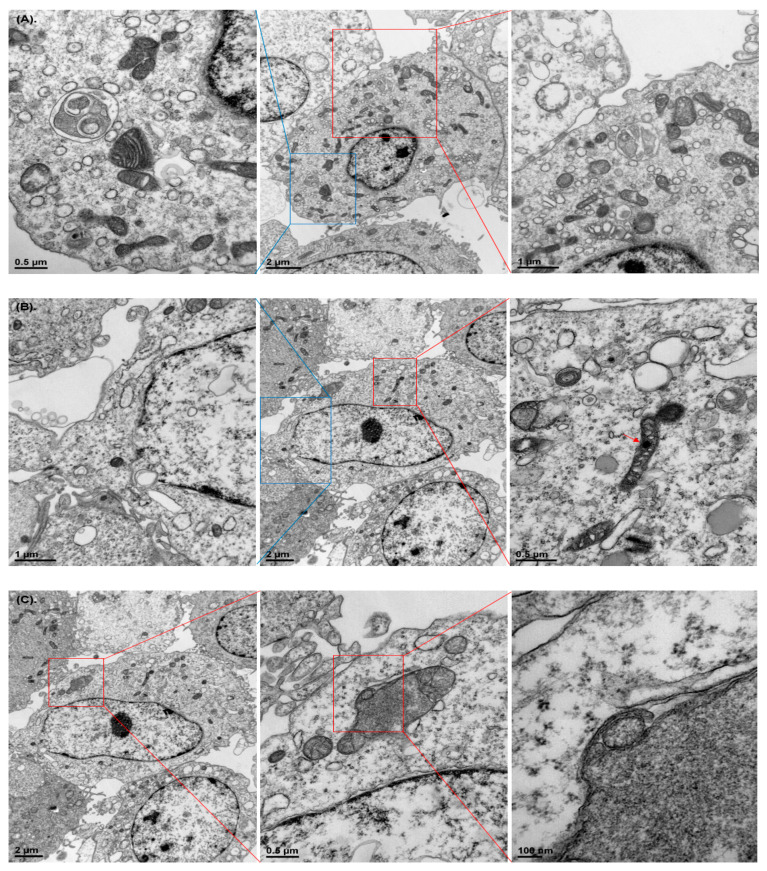
Overview of changes in intracellular structures of WCSC-treated cells. Astrocytes were incubated with 1% WCSC for 24 h, and TEM was used to observe changes in intracellular structures: (**A**) we can find autolysosome-like vacuoles (a blue square) and various sizes of mitochondria (a red square); (**B**) damage of the nuclear membrane (a blue square) and localization of WCSC components into the mitochondria (a red square, a red arrow); (**C**) mitophagy.

**Figure 4:**
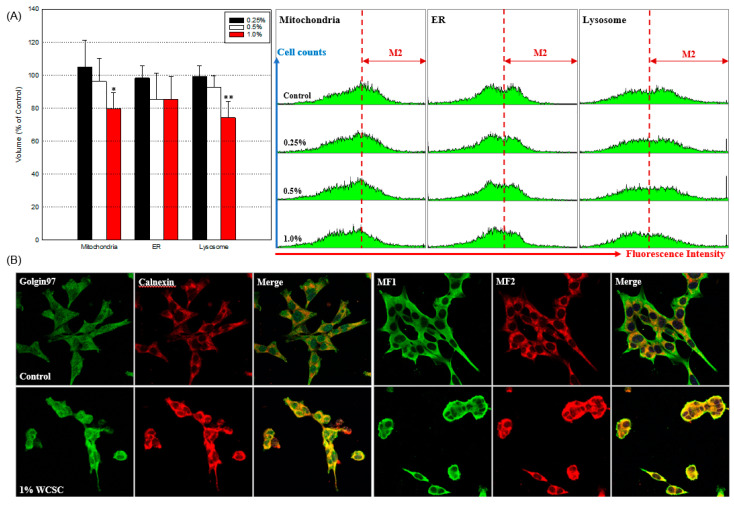
Structural damage of organelles. After 24 h exposure, astrocytes were incubated with indicators that can label each organelle: (**A**) percentages of cells in M2 zone (the increased volume). Overall, 10,000 cells per sample were analyzed using a FACS system, and the levels were calculated as the relative value compared to control (100%). The graphs on the right show the representative results. Data indicate mean ± SD of independent three experiments. * *p* < 0.05, ** *p* < 0.01; (**B**) cells were reacted with indicators for trans-Golgi (golgin97) and ER (calnexin) (left) or mitochondrial fission-related proteins (MF1 and MF2) overnight at 4 °C. Magnification: ×400.

Considering the morphological changes observed under a phase-contrast microscope, we assessed changes in intracellular structures following exposure to WCSC using TEM. We found various sizes of mitochondria, autophagosome-like vacuoles, and impaired mitochondrial structures in WCSC-treated cells (Figure 3A, Supplementary Figure S1A–C). More interestingly, damage of the nuclear membrane (Figure 3B, Supplementary Figure S1D) and phagocytosis of an organelle by mitochondria (Figure 3C) were observed in cells exposed to 1% WCSC. Furthermore, the volumes of mitochondria (79.8 ± 9.7%), ER (85.5 ± 14.0%), and lysosome (74.3 ± 10.0%) tended to decrease in cells exposed to the maximum concentration of WCSC, compared with the control (Figure 4A). Furthermore, expressions of golgin97 (an indicator for the trans-Golgi), calnexin (a marker for ER), and MF1 and MF2 (essential proteins for maintenance of mitochondrial morphology) [27] were concentrated near the nuclear (Figure 4B).

### 3.3. Mitochondrial Energy Metabolism

**Figure 5:**
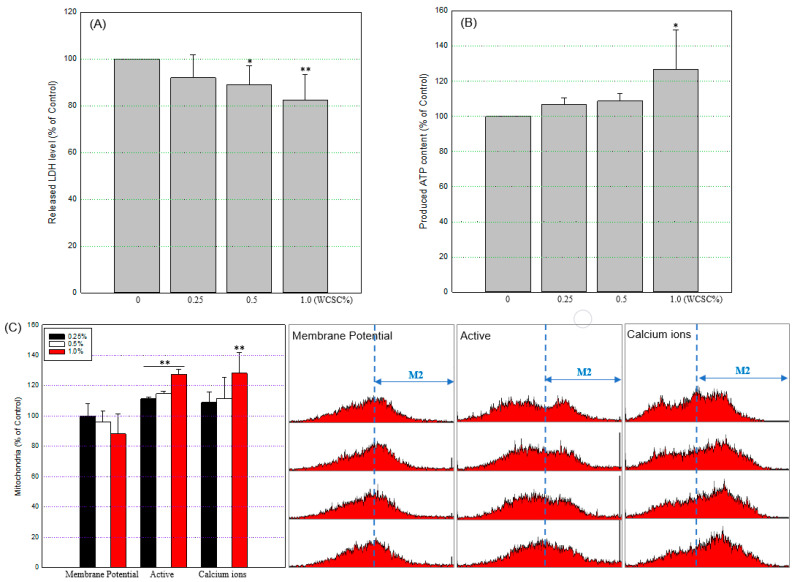
Functional damage of mitochondria. All the experiments were conducted independently three times according to the manufacturer’s instructions. * *p* < 0.05, ** *p* < 0.01: (**A**) LDH release; (**B**) produced total ATP; (**C**) mitochondria integrity. Overall, 10,000 cells per sample were analyzed using a FACS system, and the levels were calculated as the relative value, compared to control (100%). The graphs on the right show the representative results.

Cells initiate autophagic signals to maintain energy homeostasis, along with clearance of damaged organelles, and LDH catalyzes the reversible conversion of pyruvate to lactate in anaerobic conditions. In this study, the released LDH level decreased in a concentration-dependent manner, and the level was 91.9 ± 9.8%, 89.1 ± 8.1%, and 82.4 ± 10.9% of the control in cells exposed to 0.25%, 0.5%, or 1% WCSC, respectively (Figure 5A). While produced total ATP content (Figure 5B), active mitochondria (Figure 5C), and mitochondrial calcium ions (Figure 5C) tended to increase, compared to the control (126.5 ± 22.6%, 127.5 ± 3.3%, and 128.2 ± 13.6% of the control, respectively, at 1% WCSC), mitochondrial membrane potential (Δψ) tended to decrease with WCSC concentration (Figure 5C).

### 3.4. Increased Intracellular ROS

**Figure 6:**
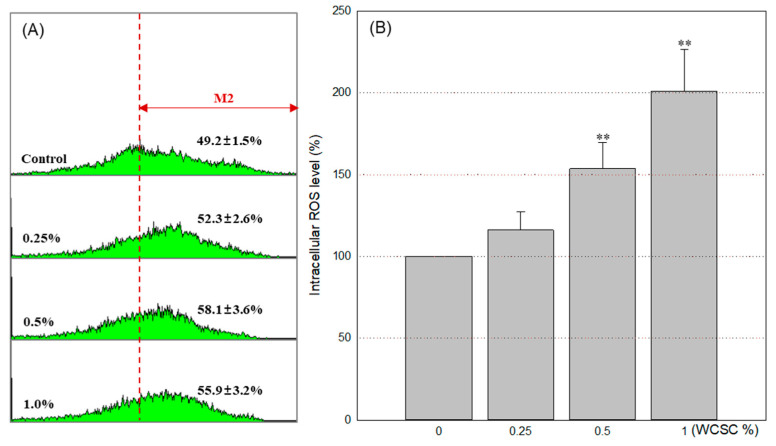
Intracellular ROS generation. Astrocytes were exposed to WCSC in designated concentrations for 24 h and then incubated with H_2_DCFDA for 1 h. Overall, 10,000 cells per sample were analyzed using a FACS system, and the levels were calculated as the relative value, compared to control (100%). Data show mean ± SD of independent three experiments. ** *p* < 0.01: (**A**) fluorescent intensity in each cell; (**B**) percentages of cells in M2 zone (cells that show increased ROS).

Given excess ROS are a key trigger of stress responses in organelles, we measured the amount of ROS generated within cells. After 24 h exposure to 0.25, 0.5, or 1% WCSC, intracellular ROS increased in a concentration-dependent manner and were 116.1 ± 11.4%, 153.6 ± 15.8% and 201.1 ± 25.3%, respectively, compared to control (Figure 6). The fraction of cells displaying increased fluorescence intensity was also clearly elevated after WCSC treatment.

### 3.5. Alteration in Gene Profiles

**Figure 7:**
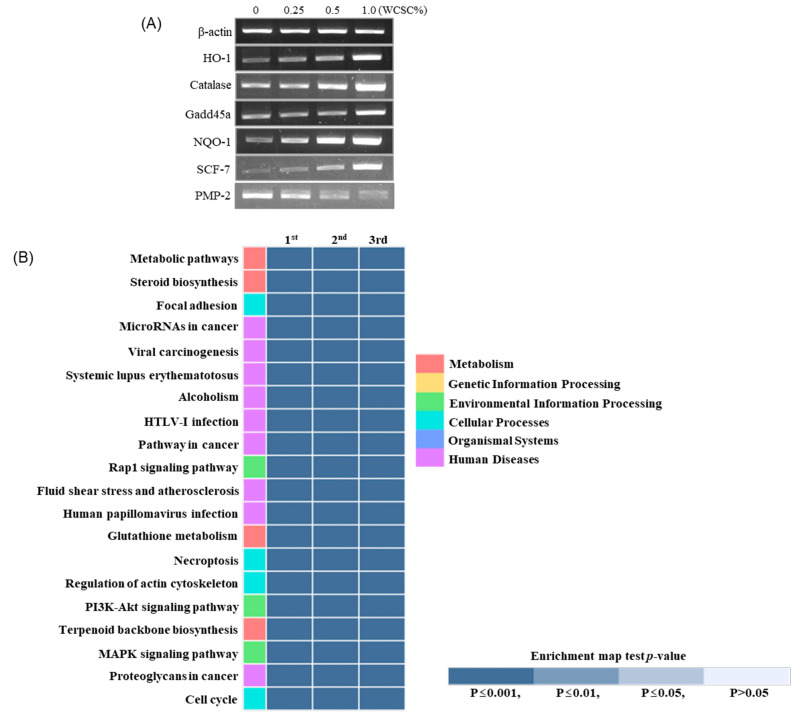
Altered gene expression: (**A**) concentration-dependent changes of individual genes. Experiments were independently performed three times. All the data showed a similar trend, and representative data were proposed; (**B**) KEGG pathway analysis. The heatmap was produced on top 20 terms in an enrichment test with *p*-value (*p* ≤ 0.001).

While the expression of genes associated with antioxidant responses (including HO-1, NQO-1, and catalase), energy metabolism (including SCF7, ATPase type 13, ATP-binding cassette, and aldehyde dehydrogenase family 3), and the immune response (including microRNA 29a, IL-11, TNF receptor, and layilin) was significantly enhanced by WCSC (1%) treatment (Table 2), expression of a gene encoding PMP2, a structural protein of peripheral nervous system myelin, was the most downregulated (Table 2). In addition, we confirmed concentration-dependent changes of some genes using the PCR analysis technique (Figure 7A). The KEGG pathway analyses also indicated that expressions of genes associated with metabolic pathways, steroid biosynthesis, and focal adhesion were the most clearly affected following exposure to WCSC (Figure 7B).

### 3.6. Accumulation of APP Protein

**Figure 8:**
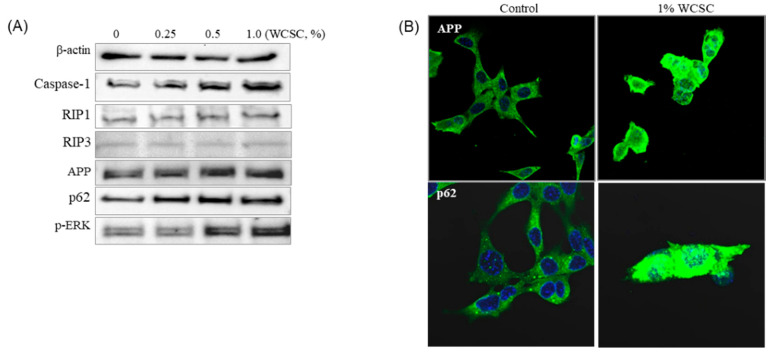
Effects on protein expression: (**A**) quantitative analysis of protein expression. Expression of all the proteins was independently tested three times, and they showed a similar trend; (**B**) visualization of the expressed protein. A p62 protein not only increased in the expression level but also seemed to be aggregated. Magnification: ×400.

The Aβ protein, a proteolytic product of APP, plays an important role in the progression of AD, and astrocytes can produce APP. In this study, we found that expression of p-ERK, p62, caspase-1, and APP proteins clearly increased in cells exposed to WCSC (Figure 8A). More importantly, fluorescence imaging showed that the specialized processes normally extending from the astrocytes were appreciably shortened or missing following exposure to WCSC (Figure 8B).

### 3.7. Disturbed Immune Response

**Figure 9:**
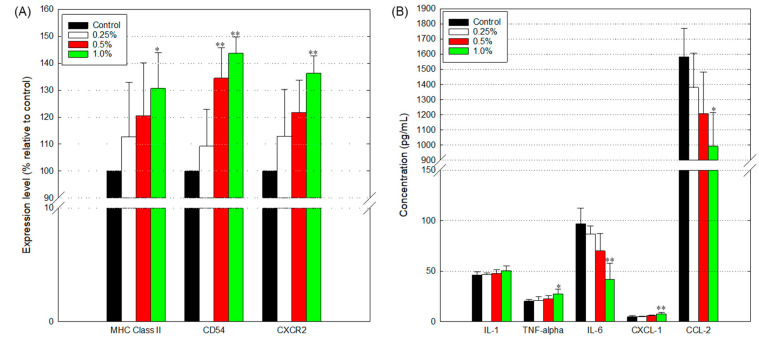
Disturbance in immune cell chemotaxis. Astrocytes were incubated with WCSC (0, 0.25, 0.5, and 1.0%) for 24 h. All the end points were independently tested three times, and the results were presented as mean ± SD. * *p* < 0.05, ** *p* < 0.01; (**A**) expression of surface molecules. Overall, 10,000 cells per sample were analyzed using a FACS system, and the surface expression level of each protein was calculated as the relative value compared to the control (100%); (**B**) concentration of cytokines (or chemokines) in the cell supernatants. The concentration was determined using two wells per sample.

Astrocytes play a central role in neuroprotective immune responses by secreting cytokines, chemokines, and growth factors. In this study, we found that expression of CD54 (an adhesion molecule), MHC class II (an essential surface molecule for the presentation of extracellular antigens), and CXCR2 (a chemokine receptor) [28] tended to be enhanced following exposure to WCSC and that the expression level 143.7 ± 6.1%, 122.7 ± 16.6% and 121.6 ± 9.5%, respectively, compared to control, on the membrane of WCSC-treated cells (Figure 9A). In addition, secretion of IL-1β, TNF-α, and CXCL-1 tended to increase following exposure to WCSC (Figure 9B). Meanwhile, the secreted IL-6 and CCL-2 level clearly decreased in the supernatants of the cells exposed to 1% WCSC (41.5 ± 16.1 pg/mL and 992.7 ± 223.8 pg/mL, respectively), compared to the control (96.8 ± 15.7 pg/mL and 1582.2 ± 190.8 pg/mL, respectively).

## 4. Discussion

Astrocytes are star-shaped cells with many processes that envelop the synapses made by neurons. As mentioned above, astrocytes are involved in essential functions, such as regulation of extracellular ion concentrations, control of synaptic function, and formation of the blood–brain barrier and cellular networks, for brain homeostasis and neuroprotection. Astrocytes also act as key regulators of neurovascular coupling, control blood flow in the brain, and participate in the clearance of harmful byproducts. Astrocytes can store glucose in the form of glycogen and also produce glucose, providing it to neurons when necessary. Based on their characteristic structures, two types of astrocytes have been identified in rodents: fibrous and protoplasmic astrocytes. While the former have only a few organelles and long processes extending from the cell body, the latter have short processes and extensive cytoplasm with abundant organelles. The processes of both types of astrocyte also have end feet that attach to the basement membrane that encircles the capillaries, helping maintain the integrity of the BBB [29,30,31]. Although the functions were not clearly elucidated, it is known that the fibrous astrocytes show a higher expression level of glial fibrillary acidic protein, an intermediate filament protein, than that in the protoplasmic astrocytes [32]. Furthermore, enhanced expression of astrocytic PMP2, a fatty-acid-binding protein that is important for the normal structure of membrane lipids, contributes to both logical thinking and cognitive functions in humans, and an increase in the diameter and number of astrocytes [33], and L1CAM is involved in neurite outgrowth and myelination, as well as cell adhesion and neuronal differentiation [34,35,36]. In this study, we used fibrous astrocytes derived from the 8-day-old mouse cerebellum, and astrocyte processes were clearly shortened following exposure to WCSC. More importantly, the expressions of PMP2 (7.9 folds) and L1CAM (4.0 folds) genes were notably downregulated in WCSC-treated cells. Therefore, we hypothesize that cigarette smoking may impair the housekeeping functions of astrocytes that maintain the structural integrity of the BBB.

The brain requires a tremendous amount of energy, and sufficient oxygen must be delivered by cerebral blood flow in order to meet this demand. In fact, although the brain comprises only 2% of the total body weight, it consumes 20% of the body’s oxygen. In addition, neurons store a limited amount of energy only, and astrocytes provide metabolic support to supply the continuous energy that neurons require. Meanwhile, the imbalance between pro-oxidants and antioxidants can result in harmful health effects, including tissue and organ damage, by inducing oxidative stress. Similarly, astrocytic oxidative stress has emerged as a critical mediator of the etiology of stroke and neurodegenerative diseases, such as AD, Parkinson’s disease, amyotrophic lateral sclerosis, and Huntington’s disease [37]. In addition, accumulating evidence indicates that metabolic disturbances and activation of inflammatory signaling pathways are closely associated with cigarette-smoking-induced diseases and that dysfunction of organelles, such as the mitochondria, ER, lysosome, and Golgi body, can be involved in different types of cell death pathways. Moreover, mitochondrial dysfunction importantly contributes to the onset of neurodegenerative diseases, and mitophagy functions as the selective scavenger toward the impaired mitochondria [38]. In our previous study, we demonstrated that WCSC contains various chemicals along with particulate materials and that it the most affected expression of iron metabolism- and cancer-related proteins, accompanied by the decrease of organelle volume and intracellular ROS and accumulation of mitochondrial calcium ions in bronchial epithelial cells [19]. Similarly, in this study, we found that the size of intracellular organelles (ER, lysosome, and mitochondria) was significantly reduced in astrocytes exposed to 1% WCSC. The mitochondrial membrane potential (Δψ) also tended to be lower in cells exposed to WCSC, and the mitochondrial calcium ion concentration was significantly elevated. In addition, the number of active mitochondria, total ATP content, and apoptotic cell death (fraction of cells in the subG1 region) increased in a concentration-dependent manner, and LDH level in the cell culture media decreased with WCSC concentrations. Additionally, WCSC clearly enhanced the expression of antioxidant-related genes accompanying a significant increase in intracellular ROS level. Moreover, TEM images indicated that the shape and size of the mitochondria were not consistent and that the cristae appeared disorganized. More importantly, phagocytosis of an organelle by mitochondria (known as “mitophagy”) was observed in 1% WCSC-treated cells, and expression of energy metabolism-related genes was clearly enhanced in the same condition. Although the role of lactate in the brain remains controversial among researchers, it is clear that lactate can be utilized as a fuel source for neurons under hypoxia conditions [39,40,41]. In addition, the balance between glycolysis and oxidative phosphorylation must be strictly controlled to maintain normal neuronal activity. Elevated ADP and calcium ion concentrations are central signals that contribute to this process, and ADP and calcium ions can depolarize the mitochondrial membrane potential [42]. Moreover, the fission of mitochondria can contribute to quality control of mitochondria through the removal of damaged mitochondria and can stimulate apoptotic cell death signals under excessive cellular stress [43]. Herein, we hypothesize that increased ATP content is attributable to an increase in the number of active mitochondria due to an imbalance between fusion and fission of mitochondria. 

Cigarette smoking has been known to contribute to the etiology of a wide spectrum of diseases, including AD. Astrocytes can also produce the Aβ, a misfolded protein found in the brain of AD patients, and incomplete clearance of produced Aβ proteins is considered an important factor in the pathogenesis of AD. Similarly, astrocytes can prevent brain damage by expressing various molecules that attract and facilitate the migration of immune cells from the bloodstream into the brain. In this study, we found autolysosome-like vacuoles and mitophagy-like phenomena in WCSC-treated cells. WCSC also increased the expression of p62, a substrate that is used as a reporter for the activation of autophagic signals [44]. Additionally, surface expression of molecules that recruit local immune cells (MHC class II, CXCR2, and CD54) was enhanced in WCSC-treated cells. Meanwhile, the appearance of dead cells (floating in the cell culture media) was not clearly observed, even in cells exposed to the highest dose. There were no notable changes in the secretion of IL-1β, TNF-α, or CXCL-1 following WCSC treatment, whereas that of IL-6 and CCL-2 was inhibited in a concentration-dependent manner. Expression of microRNAs (29a and 222) related to inhibition of autophagic signals is significantly enhanced [45,46,47], APP accumulated, together with increased expression of a p-ERK protein A p62 protein, can influence the balance of mitophagy [38], and ERK phosphorylation is a regulator of pro-inflammatory responses in AD pathogenesis [48]. Additionally, IL-6 is a pleiotropic cytokine that can act in both pro- and anti-inflammatory responses, and CCL-2 and CXCL-1 act for chemotaxis of monocytes and neutrophils, respectively [49]. Herein, we hypothesize that WCSC may cause accumulation of APP by blocking autophagic signals and contribute to the occurrence of AD due to deficiency in recruiting immune cells. 

Taken together, these results suggest that WCSC may contribute to the onset of AD by causing structural and functional damage to astrocytes. In addition, in this study, we studied the toxic response of WCSC following 24 h exposure to astrocytes. Therefore, we suggest that further study should be performed to identify the real neurotoxicity of inhaled cigarette smoke in mice.

## Figures and Tables

**Table 1:** A primer list used for PCR analysis.

Gene Name	Primer	Sequence
β-Actin	Forward	TTCTTTGCAGCTCCTTCGTT
	Reverse	CGCAGCTCATTGTAGAAGGT
Catalase	Forward	TTCGTCCCGAGTCTCTCCAT
	Reverse	GAGTGTCCGGGTAGGCAAAA
GADD45a	Forward	TGGAGGAAGTGCTCAGCAAG
	Reverse	GTCATCTCTGAGCCCTCGTG
HO-1	Forward	AACAAGCAGAACCCAGTCTA
	Reverse	CCTTCTGTGCAATCTTCTTC
NQO-1	Forward	GGTAGCGGCTCCATGTACTC
	Reverse	TGCCCTGAGGCTCCTAATCT
PMP2	Forward	AAGCCAAGAGCATCGTGACA
	Reverse	CCCGTTAGAGCGACTCATCC
SCF	Forward	GCAGACACTGGGTCTCGATT
	Reverse	GTGGTAGGGACCTTGGGTTG

**Table 2:** Gene list upregulated.

**(A) Gene List Upregulated More Than 1.6 Folds**					
**Gene_Symbol**	**mRNA Accession**	**Folds**	**Gene_Symbol**	**mRNA Accession**	**Folds**
Hmox1	NM_010442	5.85	Npc1	NM_008720	1.78
Slc7a11	NM_011990	3.90	Pgd	NM_001081274	1.77
Atp13a4	NM_001164612	3.14	Disp2	NM_170593	1.77
Cd68	NM_001291058	2.87	Sh2d6	XM_006506833	1.77
Nqo1	NM_008706	2.84	Cyb5r1	NM_028057	1.76
Abcc4	NM_001033336	2.75	Gm23302	ENSMUST00000082710	1.75
Blvrb	NM_001290525	2.74	Il34	NM_001135100	1.75
Mir29a	NR_029744	2.55	Vmn1r62	NM_030741	1.74
Gclm	NM_008129	2.44	Ampd3	NM_001276301	1.73
Gbe1	NM_028803	2.40	Adh7	NM_009626	1.73
Sox9	NM_011448	2.35	Sqstm1	NM_001290769	1.73
Gm25126	ENSMUST00000083343	2.30	Cth	NM_145953	1.72
Gm25799	ENSMUST00000082643	2.27	Cat	NM_009804	1.72
Slc40a1	NM_016917	2.25	Gm3170	ENSMUST00000168753	1.71
Gm10701	ENSMUST00000098926	2.23	Dusp5	NM_001085390	1.71
Gm25121	ENSMUST00000179846	2.20	Ptchd1	NM_001093750	1.70
Aldh3a1	NM_001112725	2.12	Lyst	NM_010748	1.69
Fbxo2	NM_176848	2.12	Bcl6	NM_009744	1.68
Tnfrsf22	NM_001311145	2.12	Sec11c	NM_025468	1.68
Il11	NM_001290423	2.12	BC048507	NM_001001185	1.67
Ypel5	NM_027166	2.11	Gtpbp2	NM_001145979	1.67
Sp140	NM_001013817	2.03	Acot2	NM_134188	1.67
Zfand2a	NM_001159908	2.02	Anxa7	NM_001110794	1.67
5330438D12Rik	XR_872054	2.00	Prdx1	NM_011034	1.67
Abcc1	NM_008576	1.99	2410006H16Rik	NR_030738	1.66
Slc48a1	NM_026353	1.98	Tbc1d30	NM_029057	1.65
Mir708	NR_030489	1.98	Gm19410	XM_006509234	1.65
Layn	NM_001033534	1.97	Uchl1	NM_011670	1.65
Btc	NM_007568	1.96	4931413I07Rik	ENSMUST00000173637	1.64
Srxn1	NM_029688	1.95	Gabarapl1	NM_020590	1.64
Sgk1	NM_001161845	1.93	Zfp2	NM_001044697	1.64
Hgsnat	NM_029884	1.93	Plk3	NM_001313916	1.64
Ptprn	NM_008985	1.89	Taldo1	NM_011528	1.63
Tenm4	NM_001310760	1.89	Clcn2	NM_009900	1.63
Gm24695	ENSMUST00000083877	1.87	Tnfaip2	NM_009396	1.63
Creg1	NM_011804	1.85	Zfp945	NM_001110254	1.63
Mllt11	NM_019914	1.85	Zfp708	NM_001012325	1.62
Mir222	NR_029807	1.85	Kdm7a	NM_001033430	1.62
Prr13	NM_001170911	1.84	Dusp4	NM_176933	1.62
LOC102638255	XR_374951	1.83	Lonp1	NM_028782	1.62
Gm25026	ENSMUST00000175017	1.82	Hid1	NM_175454	1.62
Gm24431	ENSMUST00000104073	1.81	Cdkn1a	NM_001111099	1.61
Chil3	NM_009892	1.81	Ptk2b	NM_001162365	1.61
	KnowTID_00001299	1.81	Rin2	NM_028724	1.61
Tmem158	NM_001002267	1.80	Ngf	NM_001112698	1.61
Esd	NM_001285423	1.80	Cobll1	NM_027225	1.61
Smox	NM_001177833	1.79	AA467197	NM_001004174	1.60
Fosl1	NM_010235	1.78	Clcf1	NM_001310038	1.60
**(B) Gene List Downregulated More Than 1.9 Folds**					
**Gene_Symbol**	**mRNA Accession**	**Folds**	**Gene_Symbol**	**mRNA Accession**	**Folds**
Pmp2	NM_001030305	-7.90	Vmn1r103	NM_001166737	-2.20
Gm25664	ENSMUST00000157697	-4.74	Plce1	NM_019588	-2.20
Chl1	NM_007697	-4.02	Mcam	NM_023061	-2.19
Plekhg1	NM_001033253	-3.85	Ogn	NM_008760	-2.19
Sntb1	NM_016667	-3.67	Cyp51	NM_020010	-2.18
Fndc1	NM_001081416	-3.62	Prss23	NM_029614	-2.18
Slitrk6	NM_175499	-3.50	Hist1h2ab	NM_175660	-2.18
Gm22935	ENSMUST00000103960	-3.41	Gas7	NM_001109657	-2.17
Hmgcs1	NM_001291439	-3.19	Lrrc8b	NM_001033550	-2.17
Cd200	NM_010818	-3.17	Dhrs3	NM_001172424	-2.14
Itga2	NM_008396	-3.16	Hist4h4	NM_175652	-2.13
Ly6a	NM_001271416	-3.06	Gpc4	NM_008150	-2.13
Slc35f1	NM_178675	-3.04	Epb41l4b	NM_019427	-2.11
Gm22908	ENSMUST00000177620	-2.98	Sorbs1	NM_001034962	-2.09
Gm26140	ENSMUST00000103807	-2.98	H2afx	NM_010436	-2.09
Gm23256	ENSMUST00000102235	-2.98	Dhcr7	NM_007856	-2.09
Ctgf	NM_010217	-2.97	Lgi4	NM_144556	-2.08
Has2	NM_008216	-2.87	Pls1	NM_001033210	-2.08
Adam19	NM_001291890	-2.83	Lss	NM_146006	-2.07
Rgs8	NM_026380	-2.82	Cmtm5	NM_026066	-2.07
Vgll3	NM_028572	-2.78	Prss23os	ENSMUST00000032858	-2.06
Deptor	NM_001037937	-2.75	Arhgef9	NM_001033329	-2.06
Postn	NM_001198765	-2.75	Olfr97	NM_146512	-2.05
Rassf4	NM_178045	-2.73	Acta2	NM_007392	-2.05
Plxnb3	NM_019587	-2.72	Rcan2	NM_001286653	-2.04
Ldlr	NM_001252658	-2.69	L1cam	NM_008478	-2.03
Dhcr24	NM_053272	-2.66	Hist1h2ag	NM_178186	-2.03
Igf1	NM_001111274	-2.62	Nrxn1	NM_020252	-2.02
Prelp	NM_054077	-2.62	Prex2	NM_001033636	-2.02
Gabrb3	NM_001038701	-2.62	Ptprz1	NM_001081306	-2.02
Deptor	NM_001037937	-2.61	Gm10719	ENSMUST00000099047	-2.01
Insig1	NM_153526	-2.58	Hist1h2bf	NM_178195	-2.01
Gm10801	ENSMUST00000099684	-2.53	Scn7a	NM_009135	-2.00
Idi1	NM_145360	-2.51	Serinc5	NM_172588	-2.00
Itga1	NM_001033228	-2.51	Mef2c	NM_001170537	-2.00
Fdps	NM_001253751	-2.50	Lrrc8c	NM_133897	-1.99
Fabp5	BC002008	-2.49		KnowTID_00007113	-1.98
Serpina3n	NM_009252	-2.45	Myl9	NM_172118	-1.97
Pappa	NM_021362	-2.41	Gabra2	NM_008066	-1.97
Fabp5	NM_001272097	-2.41	Hist2h2bb	NM_175666	-1.97
Rgs16	NM_011267	-2.40	Gm11168	ENSMUST00000178348	-1.97
Hist1h4n	OTTMUST00000001028	-2.37	Npnt	NM_001029836	-1.96
Ahr	NM_001314027	-2.36	Alg6	NM_001081264	-1.96
Hist1h1b	NM_020034	-2.36	Pcsk6	NM_001291184	-1.96
Iqgap2	NM_027711	-2.35	Hist1h2bn	NM_178201	-1.96
Sparcl1	NM_010097	-2.34	Fdft1	NM_010191	-1.95
Nsdhl	NM_010941	-2.34	Tgfb2	NM_009367	-1.95
Adamts1	NM_009621	-2.34	Arhgef26	NM_001081295	-1.94
Cp	NM_001276248	-2.34	Prickle1	NM_001033217	-1.94
Hist1h1a	NM_030609	-2.33	Gm10715	ENSMUST00000177969	-1.94
H1fx	NM_198622	-2.31	Msmo1	NM_025436	-1.94
Kctd12	NM_177715	-2.29	Thbs1	NM_001313914	-1.93
Nid2	NM_008695	-2.29	Stard13	NM_001163493	-1.93
Gm10717	ENSMUST00000099042	-2.29	Kcna1	NM_010595	-1.93
Ephb2	NM_001290753	-2.29	Afap1l2	NM_001177796	-1.92
Cdh19	NM_001081386	-2.27	Gm12688	XR_401838	-1.92
Tubb3	NM_023279	-2.24	Hist1h2af	NM_175661	-1.92
Rbms3	NM_001172121	-2.23	Foxd3	NM_010425	-1.91
Gm26519	XR_389454	-2.22	Nppb	NM_001287348	-1.90
Olfr344	NM_146628	-2.21	Tagln2	NM_178598	-1.90
Gm24277	ENSMUST00000158661	-2.21	Gm10717	ENSMUST00000075573	-1.90

Astrocytes were harvested after 24 h exposure with or without WCSC (1.0%) in 60 cm^2^-plates (every three plates for control and 1% WCSC), and the cells were pooled to make one sample for analysis. Samples were independently prepared three times, and tables show genes that are changed with statistical significance, and data are the mean value.

## Data Availability

The data presented in this study are available on request from the corresponding author.

## Supplementary Materials

The following are available online at https://www.mdpi.com/article/10.3390/toxics9070150/s1, Figure S1: TEM images of cells exposed to 1% WCSC.
