# Supplementary material for: Whole Cigarette Smoke Condensates Induce Accumulation of Amyloid Beta Precursor Protein with Oxidative Stress in Murine Astrocytes
Source: Toxics. 2021 Jun 28;9(7):150. doi: 10.3390/toxics9070150 (PMC8309752; doi:10.3390/toxics9070150)
Supplement: Supplementary file 1 [file toxics-09-00150-s001.zip › toxics-1250437-supplementary.pdf]

Supplementary Materials: Whole cigarette smoke condensates induced accumulation of amyloid beta precursor protein with oxidative stress in murine astrocytes

Eun-Jung Park, Seung-Woo Jin, Hyun-Ji Lim, Hyeon-Young Kim, **Min-Sung Kang,**

Siyoung Yang

(A)

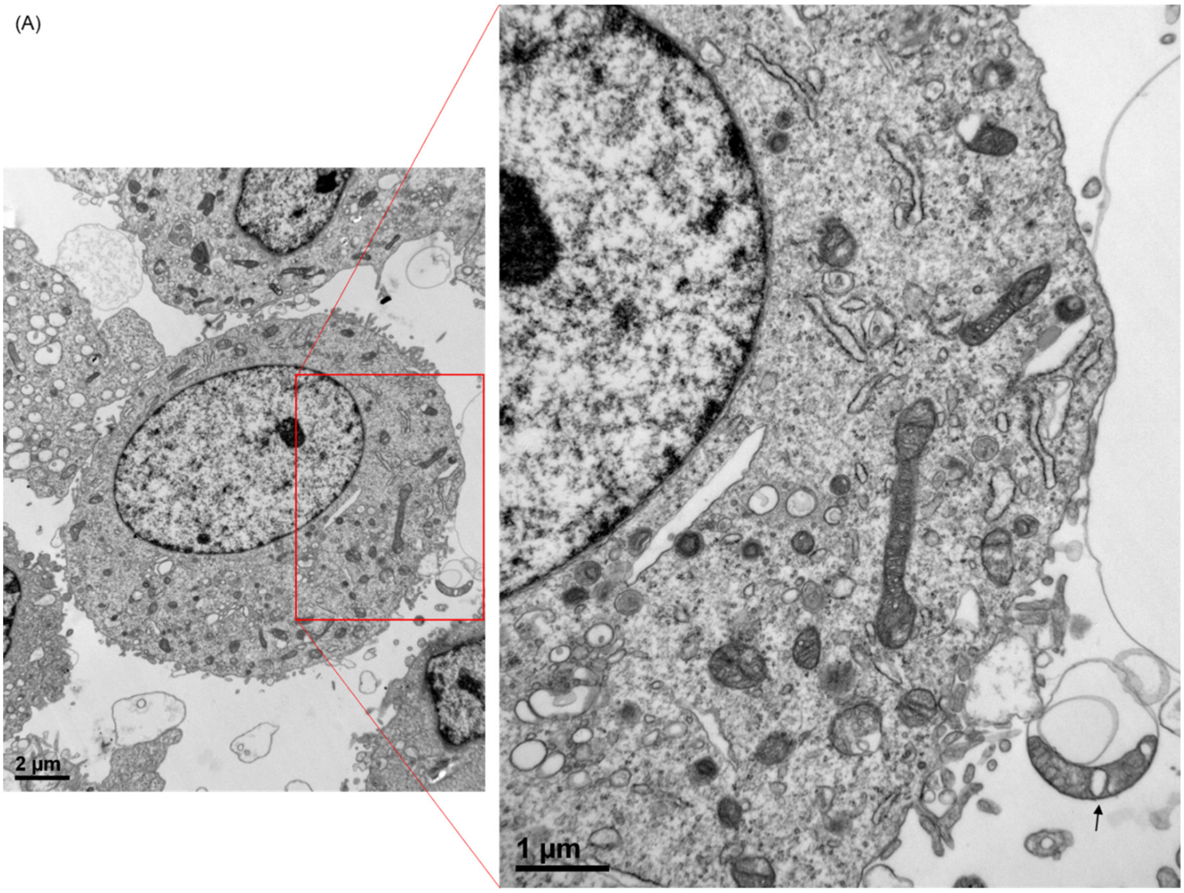

(B)

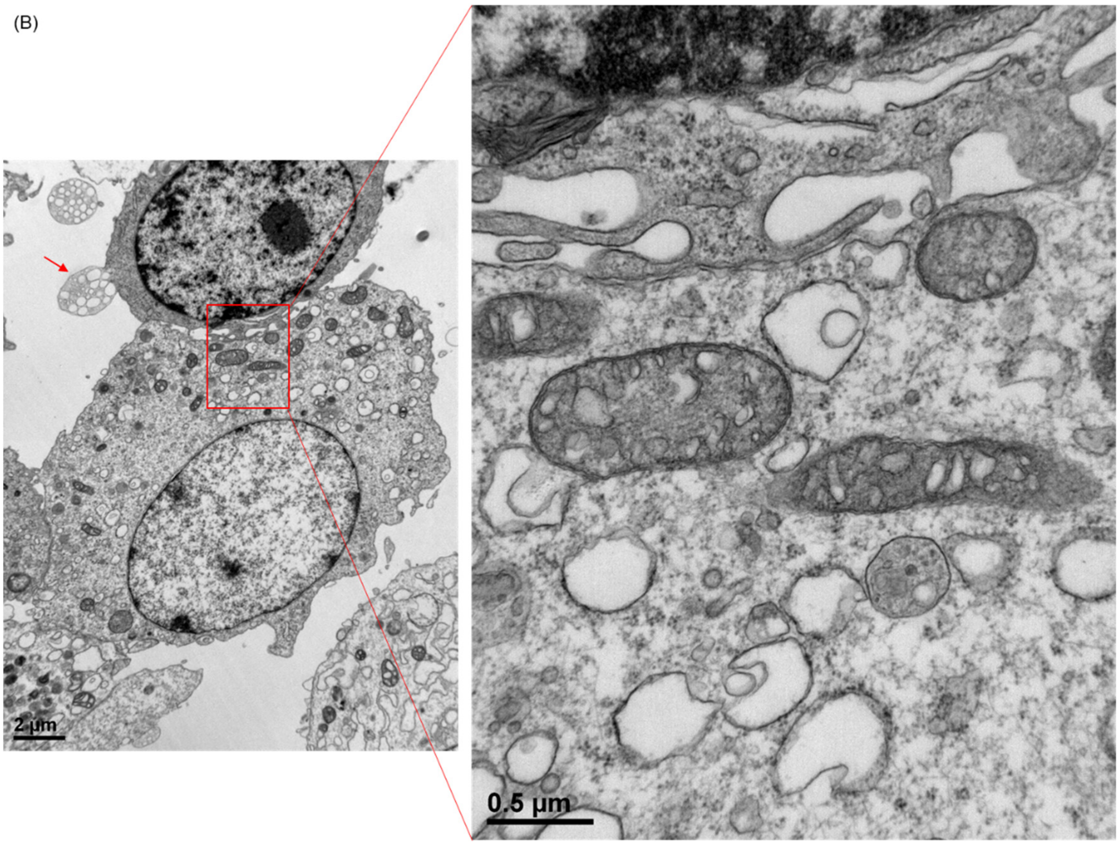

(C)

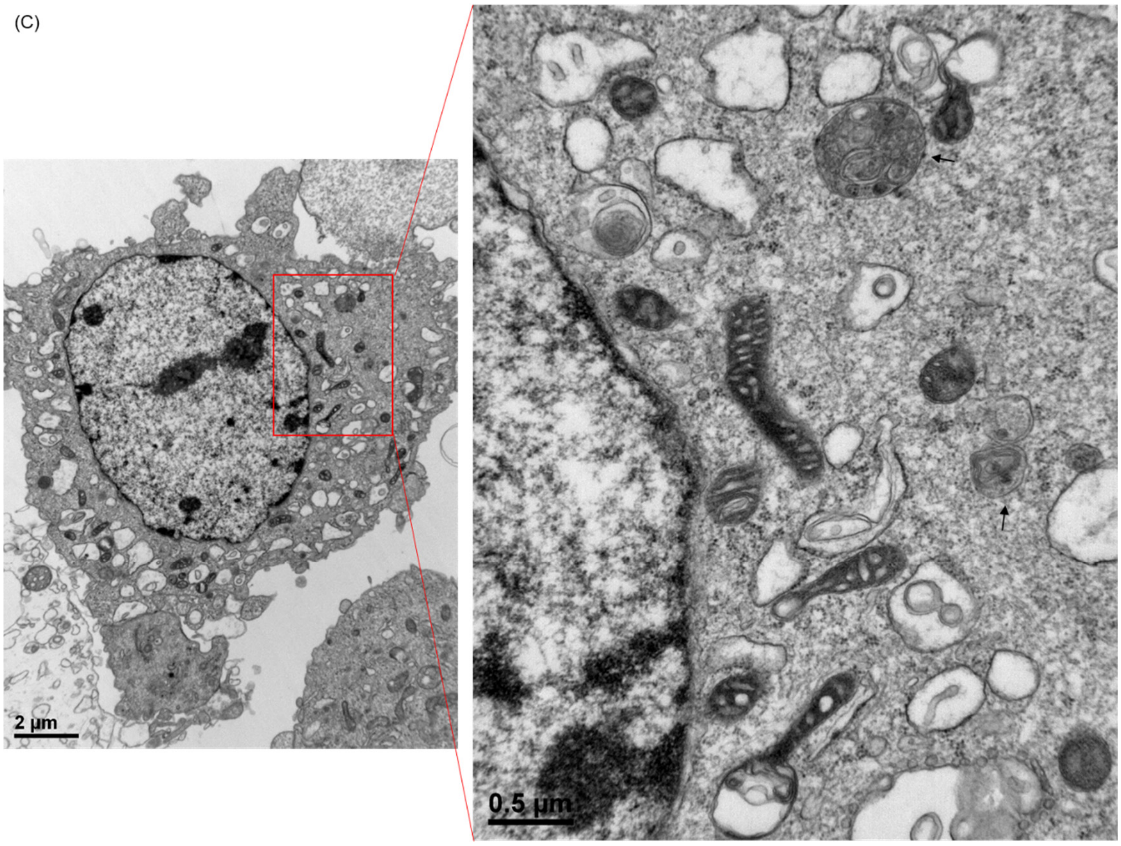

(D)

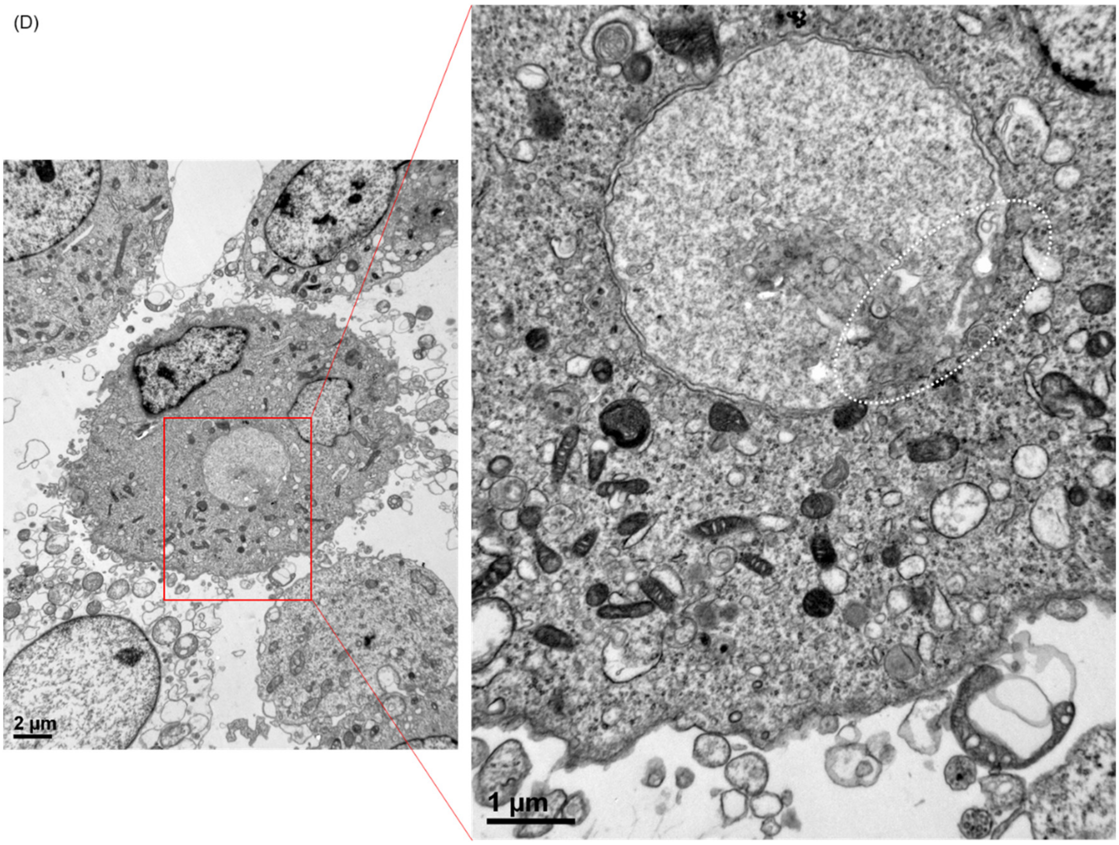

Figure S1. TEM images of cells exposed to 1% WCSC. We could find mitochondria impaired structurally in (**A**), (**B**) and (**C**). In addition, extracellular mitochondria (A, a black arrow), exocytosis of a vacuole containing damaged organelles (B, a red arrow), autolysosom-like vacuoles (C, white arrows), and the nuclear membrane collapse (D, a white dotted line circle) were observed.
